# Elastin-Like Protein, with Statherin Derived Peptide, Controls Fluorapatite Formation and Morphology

**DOI:** 10.3389/fphys.2017.00368

**Published:** 2017-06-08

**Authors:** Kseniya Shuturminska, Nadezda V. Tarakina, Helena S. Azevedo, Andrew J. Bushby, Alvaro Mata, Paul Anderson, Maisoon Al-Jawad

**Affiliations:** ^1^Dental Physical Sciences Unit, Institute of Dentistry, Barts and The London School of Medicine and Dentistry, Queen Mary University of LondonLondon, United Kingdom; ^2^School of Engineering and Materials Science, Queen Mary University of LondonLondon, United Kingdom; ^3^Materials Research Institute, Queen Mary University of LondonLondon, United Kingdom; ^4^Institute of Bioengineering, Queen Mary University of LondonLondon, United Kingdom

**Keywords:** enamel biomimetics, elastin-like proteins, fluorapatite, biomineralization model

## Abstract

The process of enamel biomineralization is multi-step, complex and mediated by organic molecules. The lack of cells in mature enamel leaves it unable to regenerate and hence novel ways of growing enamel-like structures are currently being investigated. Recently, elastin-like protein (ELP) with the analog *N*-terminal sequence of statherin (STNA15-ELP) has been used to regenerate mineralized tissue. Here, the STNA15-ELP has been mineralized in constrained and unconstrained conditions in a fluoridated solution. We demonstrate that the control of STNA15-ELP delivery to the mineralizing solution can form layered ordered fluorapatite mineral, via a brushite precursor. We propose that the use of a constrained STNA15-ELP system can lead to the development of novel, bioinspired enamel therapeutics.

## Introduction

It is well established that enamel biomineralization occurs via a complex, multistep and matrix mediated process. The matrix is made up of many macromolecules including glycoproteins and proteins rich in negatively charged residues, such as amelogenin, enamelin and amelotin (Iijima et al., 2010; Moradian-Oldak and Paine, 2010; Abbarin et al., 2015). These negative residues can bind calcium ions, initiate nucleation and, *via* inhibition and promotion, carefully regulate the crystal growth, morphology and alignment (Mann, 2001). Specifically, biomineralizing proteins have stereochemical matching to crystal surfaces and can inhibit crystal growth in a particular direction. The crystal growth process is further controlled by local pH changes that are said to be crucial in normal enamel development (Lacruz et al., 2010).

The process of dental enamel formation is tightly controlled by ameloblasts and produces a highly organized mineral structure that protects us from infection and mastication forces. However, enamel becomes completely acellular and almost depleted of the proteinaceous matrix when mature. During enamel maturation, the process of enzymatic protein degradation and removal is essential in order to allow for the apatite crystals to fill up the space. The end result is a highly mineralized tissue with a high modulus and hardness arising from 96% by weight mineral content (Deakins and Volker, 1941; Cuy et al., 2002). Although enamel's structure and composition give it its superior mechanical properties, the lack of cells makes it unable to biologically regenerate following damage.

Materials have been developed and used in order to restore enamel tissue when it has been lost after carious infection, removal or trauma. However, these materials lack the intrinsic hierarchical structure and in general have inferior properties compared to enamel, leading to further failure. Today, researchers are looking for new and innovative ways of forming enamel-like structures for repair or remineralization of early carious enamel lesions. Examples of these include the formation of enamel prism-like bundles of apatite needles synthesized in solution (Chen et al., 2005), the use of casein for remineralization (Vashisht et al., 2010), use of a modified hydroxyapatite paste (Yamagishi et al., 2005) and synthetic or natural peptides which promote apatite formation (Brunton et al., 2013; Ruan and Moradian-Oldak, 2014). These examples provide evidence of ordered hydroxyapatite (HAp) formation, epitaxial growth or remineralization.

Often, research looks at including fluoride ions (F^−^) into therapeutics such as toothpastes. The F^−^ ions can incorporate into the apatite structure by replacing the hydroxide ion (OH^−^) located in the middle of the hexagonal HAp crystal. This exchange forms fluorapatite (FAp). The F^−^ causes a reduction in the *a* and *b* unit cell parameters of the apatite structure which directly decreases the crystal energy (Robinson et al., 1995). The reduced crystal energy gives rise to the increased stability of FAp in acids, compared to HAp, deeming it more useful in dental applications. FAp has been synthesized in a variety of ways, for example as a coating on implants (Czajka-Jakubowska et al., 2009; Dunne et al., 2015) or hydrothermally in solution (Chen et al., 2006). However, in order to mimic the natural biomineral formation, we must understand how proteins can be utilized in order to control the nucleation, growth and morphology of FAp.

Recently, elastin-like proteins (ELPs) have been exploited for use in synthetic biomineralization. These are recombinant proteins, produced by genetically modified bacteria, that can be engineered to contain various bioactive sites (Girotti et al., 2004). One such bioactive site is the analog of the 15 amino acid *N-*terminal of statherin (STNA15). Statherin, a 43 amino acid protein present in saliva, aids in remineralization of enamel, on a daily basis, via calcium ion chelation. It is believed that statherin's high affinity to apatite arises from its acidic *N*-terminal domain (Raj and Johnsson, 1992). However, more recent work showed that the basic residues, such as arginine and lysine, also play a crucial role in the interaction of statherin with an apatite surface (Ndao et al., 2010). In the STNA15 sequence, the phosphorylated serines (positions 2 and 3), present in natural statherin, are replaced with aspartic acid (Raj and Johnsson, 1992), removing the need for post-translational modification and still retaining the calcium affinity. Elastin-like protein containing the statherin derived peptide sequence (STNA15-ELP) membranes have already shown potential in bone repair (Tejeda-Montes et al., 2014). Furthermore, we have previously demonstrated the ability of these ELPs to form organized apatite structures (Elsharkawy et al., 2016a,b). The focus of the study presented here is to extend the use of the STNA15-ELP to enamel therapeutics. Here, we utilize the ELPs with the STNA15 sequence in order to study their ability to promote organized fluoridated calcium phosphate formation. The role of STNA15-ELP in different conditions is explored by comparing the effect of mineralizing the protein directly in solution or immobilized on a glass surface.

## Materials and methods

### STNA-15 ELP

The STNA15-ELP was acquired from Technical Proteins Nanobiotechnology (Valladolid, Spain). These recombinant proteins are produced by genetically modified bacteria and extracted at the company (Girotti et al., 2004). The full sequence of the protein used in this study is MESLLP-[((VPGIG)_2_VPGKG(VPGIG)_2_)_2_-**DDDEEKFLRRIGRFG**-((VPGIG)_2_VPGKG(VPGIG)_2_)_2_]_3_-V where the STNA15 sequence is highlighted in bold. It has an inverse transition temperature (ITT) of 23°C and isoelectric point (pI) at pH 9.9 (details provided by the supplier).

To prepare the stock protein solution, 1 mg of STNA15-ELP was dissolved in 1 ml of ultrapure water (18 MΩ·cm). The stock solution was then diluted to yield a 100 μg/ml concentration. To prepare constrained protein samples, 100 μl of the dilute solution was pipetted onto borosilicate glass slides (VWR International Ltd, Lutterworth, UK) and left to completely dry at 21°C overnight.

To prepare the unconstrained STNA15-ELP samples, 100 μl of the 1 mg/ml protein stock solution was pipetted directly into 1 ml of the mineralizing solution. After the mineralization period of either 3 h or 8 days, the samples were frozen in liquid nitrogen and lyophilized. The lyophilized samples were washed with ethanol and dried prior to chemical and morphological analysis.

### Mineralizing solution and studies

The mineralizing solution was used as previously described by Chen et al. (2006). In short, 104.7 mg sinter grade HAp (Plasma Biotal Ltd., Derbyshire, UK) and 8.49 mg sodium fluoride (Sigma Aldrich, UK) was added to 100 ml of ultrapure water (18 MΩ·cm). Sixty nine percent analytical grade nitric acid (VWR International Ltd., Lutterworth, UK) was added drop-wise until the solution became clear and colorless and a pH of 2.4 was reached. Twenty eight to thirty percent ammonium hydroxide (Sigma Aldrich, UK) was added drop-wise to the solution until pH of 6 was reached. The solution was prepared at room temperature and stirred continuously during preparation.

Three milliliters of solution were incubated with each coated sample and 1 ml with STNA15-ELP solution samples. For the coated samples, uncoated borosilicate glass was incubated in the mineralizing solution as a control. Precipitate from pure mineralizing solution, without any protein, was used as a control for unconstrained protein samples. All samples were incubated in the mineralizing solution for either 3 h or 8 days at 37°C in sealed containers.

### Quartz crystal microbalance (QCM)

Quartz crystal microbalance measurements were carried out in order to check the affinity of the protein to the borosilicate glass. Borosilicate coated quartz sensor crystals (Biolin Scientific Ltd, Stockholm, Sweden) were first washed in a 2% w/v SDS (Sigma Aldrich, UK) solution for 30 min followed by a 10 min UV/ ozone treatment as a cleaning procedure. In the QCM (Q-sense, Biolin Scientific Ltd, Stockholm, Sweden) the crystal was stabilized in ultrapure water then, a solution of STNA15-ELP (100 μg/ml) was added and the QCM measurement was taken until equilibrium was reached. Finally, the crystal was washed again with ultrapure water to remove any unbound or loosely bound protein. The change in resonant frequency of the quartz crystal was converted to mass of protein adsorbed using the Sauerbery equation (Equation 1) where Δ*m* is the change in mass, Δ*f* the measured change in frequency upon protein adsorption, *n* is the overtone number (3) and C is a constant specific to the crystal.

(1) $$\begin{array}{r} \Delta m=-\frac{C.\Delta f}{n} \end{array}$$

### Contact angle

A DSA100 Drop Shape Analyzer from Krüss (Hamburg, Germany) was used to measure the contact angle of water on uncoated and coated borosilicate glass slides. Five microliters of water were pipetted onto the slide and the angle measured immediately. Three repeats were taken and averaged.

### Scanning electron microscopy (SEM /energy dispersive X-ray analysis (EDX)

Scanning electron microscopy (SEM) images were recorded, using a secondary electron (SE) and back-scattered electron (BSE) detectors, on an FEI Inspect F SEM (Hillsboro, Oregon, USA) in the NanoVision Centre, Queen Mary University of London. An X-Act Oxford Instruments EDX detector was used for EDX measurements (20 kV accelerating voltage) (Abington, Oxfordshire, UK). The samples were coated with carbon for EDX studies and 20 nm of gold for morphological examination.

### Transmission electron microscopy (TEM)/selected area electron diffraction (SAED)

TEM images and SAED patterns were recorded on a JEOL 2010 transmission electron microscope operated at 200 kV (Tokyo, Japan). For the 3 h incubation timepoint of constrained ELP mineralization, the crystals were grown directly on a TEM grid. The 8 day mineralized samples were scraped off the borosilicate glass into ethanol and then pipetted onto grids.

The 3 h and 8 day unconstrained samples were first suspended in ethanol then pipetted onto copper grids and dried prior to TEM inspection.

### Attenuated total reflection fourier transform infrared spectroscopy (ATR-FTIR)

ATR-FTIR was carried out using a Bruker Tensor 27 IR spectrometer (Billerica, Massachusetts, USA) to analyze both protein conformation and chemical groups of the mineral precipitate. STNA15-ELP was prepared as previously described with the ultrapure water exchanged for deuterium oxide (D_2_O) (VWR International Ltd, Lutterworth, UK).

Before FTIR measurement, an STNA15-ELP coated glass slip was rinsed with D_2_O to remove excess protein. The measurements on the coating were taken in a wet condition. Free protein samples were pipetted directly on the ATR window. Eighty scans per measurement, 400–2,000 cm^−1^ range, 3 repeats were acquired and averaged. The FTIR chamber was purged with nitrogen during readings. The amide I region was deconvoluted in Origin Pro using the Gaussian fit and previously reported literature values (Serrano et al., 2007). For the characterization of the mineral precipitate, 60 scans, in the range of 400–4,000 cm^−1^, were taken for each mineralized sample. All measurements were carried out at 21°C.

### Raman spectroscopy

Since the FTIR spectrum of borosilicate glass has large absorptions in the range where calcium phosphate peaks are present, Raman spectroscopy was chosen to analyze the mineral grown on constrained ELP surfaces. A Renishaw inVia Raman Microscope (Wotton-under-Edge, Gloucestershire, UK), equipped with a 633 nm wavelength (20 mW power) laser, was used to record the spectra of the mineral formed in the presence of constrained STNA15-ELP at 3 h and 8 days. A 20x objective was used, giving a spot size of 1.93 μm. The spectra were obtained in the 170–1370 cm^−1^ range with an exposure time of 5 s. Sixty accumulations were recorded for each reading.

## Results and discussion

### STNA15-ELP adsorption and conformation

**Figure 1:**
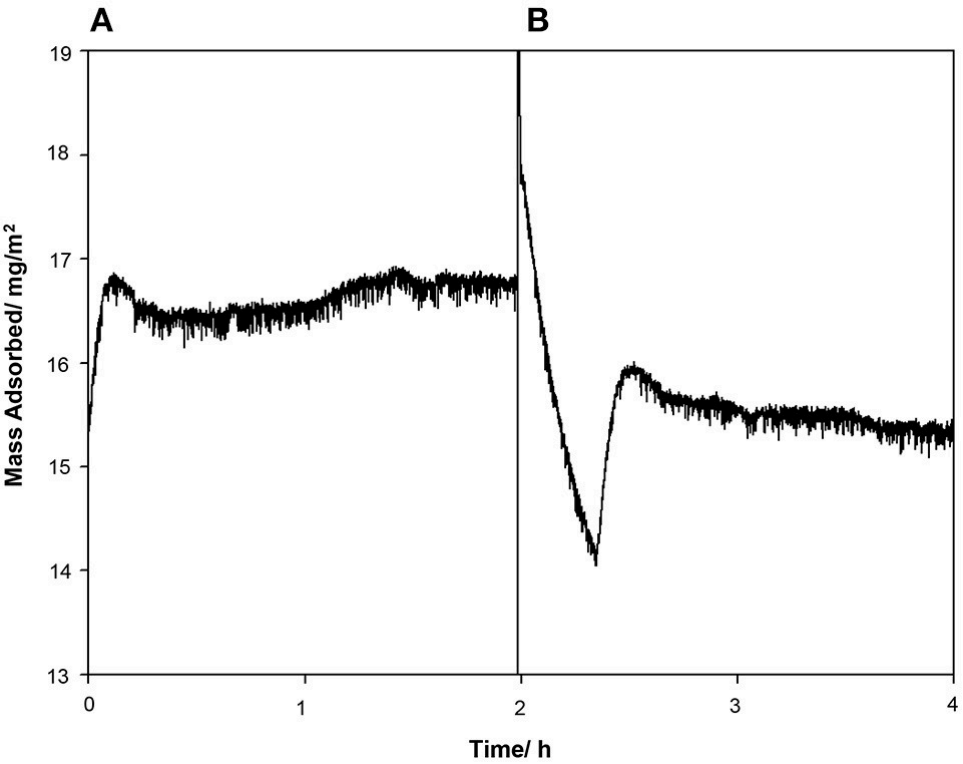
QCM data showing the **(A)** protein injection and **(B)** washing cycles. After the washing cycle the final measured mass adsorbed to the surface was 15.2 mg/m^2^.

The contact angle of the borosilicate glass surface displayed in Table 1 is indicative of a surface with low hydrophilicity (θ = 70.9°C). Reports have shown that protein adsorption is favored when θ > 65°C (Vogler, 2012). Under the given experimental conditions, according to literature value for the isoelectric point (pI), the glass carries a net negative charge (Blass et al., 2013). The protein can arrange itself on the glass surface in such a way that the negative STNA15 (mineralizing) sequence is exposed to the solution due to repulsion from the negatively charged glass surface. The positively charged lysine residues in STNA15-ELP are expected to interact with the negative glass surface. The QCM study confirmed that the STNA15-ELP, in ultrapure water, does bind to the borosilicate glass. The study showed a good binding of STNA15-ELP where 15.2 mg/m^2^ remained adsorbed to the substrate after washing with ultrapure water (Figure 1B). The contact angle of the coating suggests that the STNA15-ELP coating formed a hydrophilic surface when adsorbed to the glass (θ = 18.7°C, Table 1), suggesting the exposure of the hydrophilic mineralizing sequence on the glass surface.

**Table 1:** Contact angle measured for ultrapure water on uncoated borosilicate glass substrate and on a protein coated borosilicate glass (*n* = 3) are given accompanied with the standard error (σ).

**Borosilicate glass property**	**Value ± σ**
Water contact angle/θ (experimental)	70.9° ± 2.0
Water contact angle/θ of protein coated glass (experimental)	18.7° ± 1.25
Isoelectric point/pH	3.62 ± 0.12

*The value for the isoelectric point of the glass was obtained from literature (Blass et al., 2013)*.

**Figure 2:**
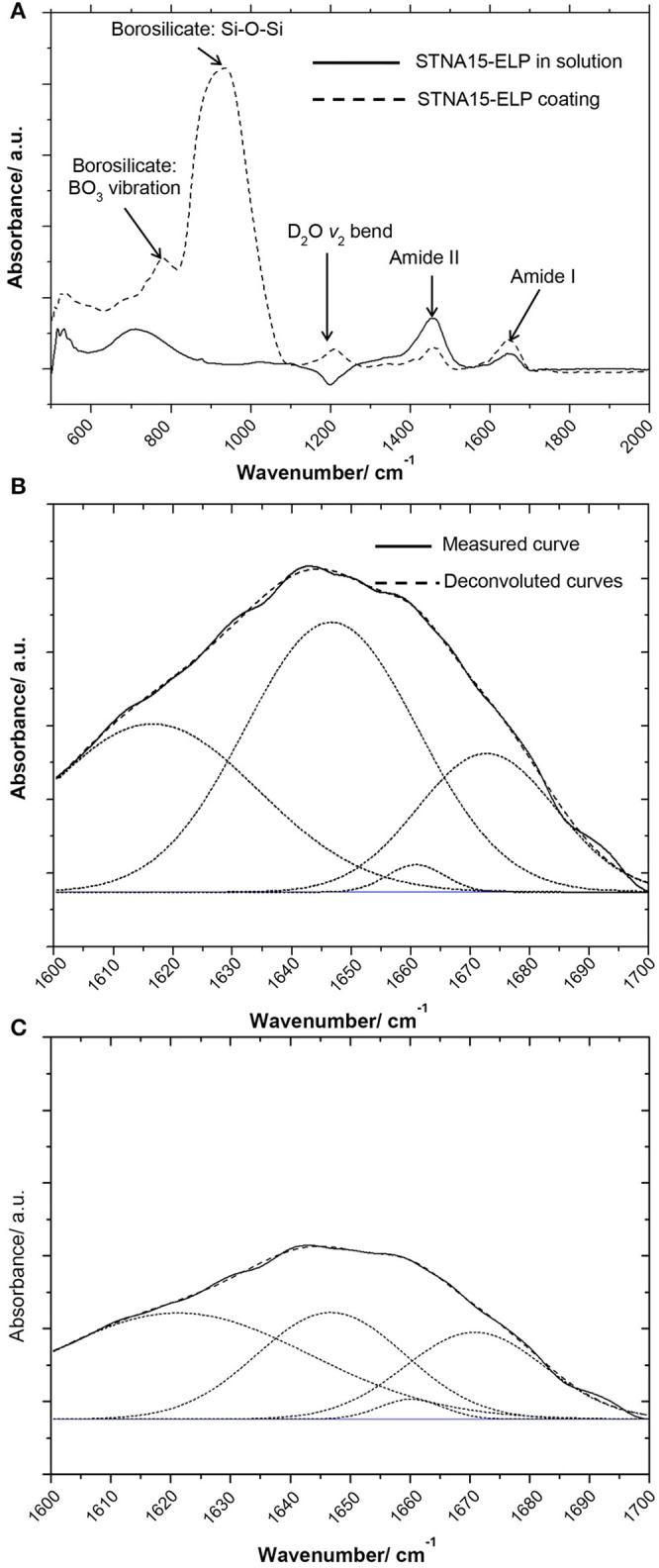
FTIR results of **(A)** coated ELP (dotted line) vs. STNA15-ELP in solution (solid line) showing the difference in the absorbance, especially noticeable difference in the intensity of amide I and II peaks. The Gaussian fit deconvoluted amide I band of **(B)** STNA15-ELP coated on glass and **(C)** protein in solution. The dashed line represents the data and the smooth line is the Gaussian fit.

The FTIR spectra of STNA15-ELP in solution vs. STNA15-ELP coating are shown in Figure 2. There is a noticeable difference in the amide I (1,600–1,700 cm^−1^) and amide II (1,400–1,500 cm^−1^) relative intensities (Figure 2A). The amide peaks are normally made up of several components arising due to the stretching vibration of C = O (amide I) and C-N and N-H bending (amide II) (Lenk et al., 1991). The position of the peaks changes depending on the secondary structure of the protein as the hydrogen bonding within the protein alters upon adsorption and/or conformational changes. The dipole moments of the amide I and amide II peaks are almost perpendicular to one another (Miyazawa and Blout, 1961), hence the change in amide I and amide II intensity is consistent with differences in conformation between the bound and unbound proteins. The overall amide I peak increased in intensity when STNA15-ELP was adsorbed on glass, conversely there was a reduction in the amide II intensity, compared to STNA15-ELP prior to adsorption (Figure 2A). Conformational changes of the ELP, upon binding, agree with previous reports of natural elastin structural changes during surface adsorption. Natural elastin is globular in solution and undergoes conformational changes in order to assemble into an ordered structure upon surface adsorption (Subburaman et al., 2006).

To investigate further, the solution (Figure 2B) and coated (Figure 2C) samples' amide I peaks were deconvoluted using a Gaussian fit. The peak values were chosen according to previous indexed values in literature (Serrano et al., 2007). The results indicate a relative increase in the amount of β-sheet/β-turn structure (at 1,647 cm^−1^), when the protein was constrained on glass, accompanied with a relative decrease in the random coil component (at 1,660/1,661 cm^−1^) (Table 2). The increase in the amount of β-sheet/β-turn structure and a decrease of the random coil in the amide I band is indicative of an increase in conformation of the secondary structure of STNA15-ELP when adsorbed on a glass substrate.

**Table 2:** Wavenumber values of the peak centers and the % area of each assigned peak taken from the deconvolution of the amide I peaks of STNA15-ELP coating and in solution (plotted in Figure 2).

**Structure**	**Constrained STNA15-ELP**		**STNA15-ELP in solution**	
	**Peak center/cm^−1^**	**Area/%**	**Peak center/cm^−1^**	**Area/%**
β-sheet aggregation	1,617	30.2		
β-sheet			1,621	44.3
β-sheet/β-turn	1,647	48.4	1,647	30.2
Random coil	1,661	1.6	1,660	2.4
β-turn	1,673	19.7	1,671	23.1

ELPs are known to have thermoresponsive properties, attributed to their ITT (Urry et al., 1998). It is known that below the ITT the protein is unfolded, mostly with a random coil structure, and the hydrophobic residues are hydrated with clathrate-like water structures (Rodriguez-Cabello, 2004). Above this temperature, the ordered water molecules surrounding the protein are disrupted and the protein becomes dehydrated. The dehydration process causes the protein to fold, via an increase in the β-sheet/β-turn component and simultaneous decrease in random coil, and phase separate. This process is not instantaneous and structural changes occur over a range of temperatures above and below the ITT (for example Reiersen et al., 1998). Previous work by Serrano et al. (2007) investigated ELP structures with FTIR and concluded that below the ITT the protein contains the β-sheet aggregation component (1,616 cm^−1^), but this completely disappears upon heating above the ITT. The FTIR data presented here shows a similar change between the unconstrained and the constrained STNA15-ELP. From the results presented in Figure 2 and Table 2, it can be expected that upon adsorption on the glass surface the ELP folds into a similar conformation as it would when heated well above the ITT, i.e., the protein loses the random coil component and gains order in its secondary structure.

Protein adsorption is a complex process and can change dramatically with alteration in the surface and solution chemistry. A number of factors that affect adsorption of proteins onto surfaces have been identified. These include pH (pH close to pI promotes adsorption) (Norde, 1986; Norde et al., 1986); substrate hydrophobicity (Shirahama and Suzawa, 1985); dehydration (Lee and Ruckenstein, 1988); and surface-solution equilibrium (Wojciechowski et al., 1986). Although protein adsorption mechanisms can be the result of multiple factors, the adsorption behavior is specific to each protein and the surface it is adsorbing to. In this study, the STNA15-ELP is dissolved in ultrapure water (pH ~7.0) and is far from its pI. Thus, according to Norde et al. (1986), it would not favorably adsorb to the glass. However, the QCM results (Figure 1) confirm that the protein is well bound to the glass surface. Lee and Ruckenstein (1988) proposed that proteins gain entropy upon adsorption to surfaces due to dehydration and structural changes. This explanation is more suitable for the system described here, since STNA15-ELP appears to gain conformation upon adsorption and this is known to occur during the dehydration of the ELP chains and subsequent hydrophobic collapse.

### Mineral morphology and chemistry

#### Constrained STNA15-ELP

**Figure 3:**
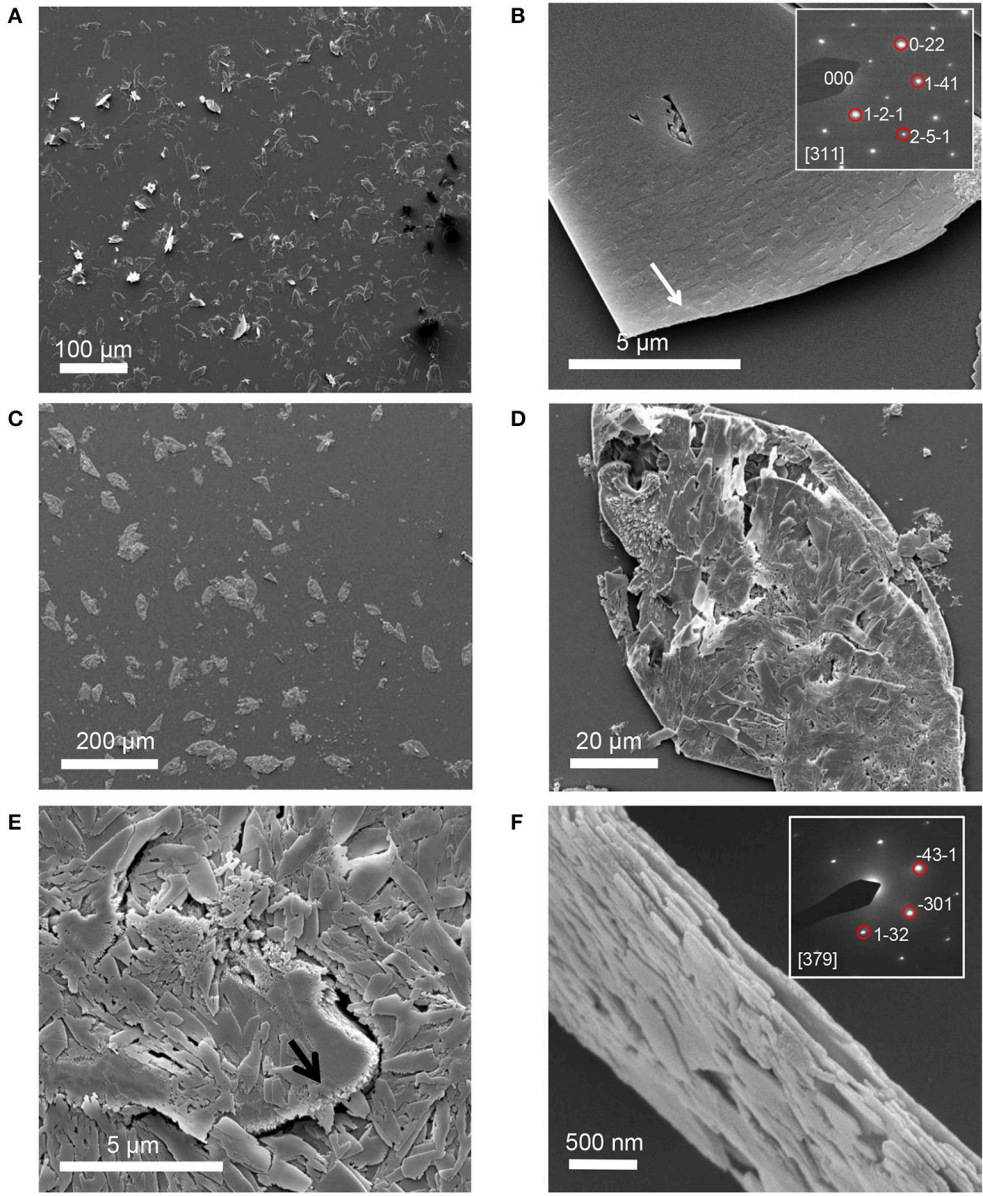
SE images of mineral grown on an STNA15-ELP coated substrate at 3 h **(A,B)** and 8 days **(C–F)**. Single crystal platelets dominate the surface at 3 h with the SAED pattern of brushite, inset of **(B)**. The platelets are polycrystalline FAp at 8 days of incubation. SAED pattern of one of the crystals from the platelet, inset of **(F)**.

STNA15-ELP coated on borosilicate glass, incubated in the mineralizing solution at 37°C, produced mineral platelets at both 3 h and 8 days incubation times, as shown in Figures 3A–F respectively. EDX analysis (3 hC, Table 3) suggests that the platelets at 3 h were brushite [dicalcium phosphate dihydrate (DCPD)], with a Ca/P ratio of approximately 1. Brushite presence was confirmed with SAED (inset Figure 3B). Etch pits were visible on the brushite surface due to dissolution of the crystal (arrow, Figure 3B). A previous study reported plate-like brushite to grow with a dominant face in the {010} direction (Giocondi et al., 2010). The etch pits typically form on the {010} faces of the brushite crystals. The platelets at day 8 retained their overall platelet shape (Figure 3D) but appeared to be a polycrystalline material. EDX analysis of the 8 day sample indicated that the platelets were fluorapatite, with a stoichiometric Ca/P ratio of 1.66 (8 dC, Table 3). The SAED of platelets present at 8 days was also indicative of the presence of apatite (inset of Figure 3F), agreeing with the EDX analysis.

**Table 3:** EDX data showing the atomic % of elements present in each sample.

**Element**	**Atomic %**				
	**Constrained STNA15-ELP**		**Free STNA15-ELP**		**CaF_2_**
	**3 hC**	**8 dC**	**3 hS**	**8 dS**	
F	–	29.6	38.6	27.9	51.1
P	48.1	26.5	24.7	28.3	2.8
Ca	51.9	43.9	36.7	43.8	46.1
Ca/P Ratio	0.93	1.66	1.48	1.55	16.56

*3 hC and 8 dC samples were incubated with the protein adsorbed on the glass for 3 h and 8 days respectively. 3 hS and 8 dS samples were incubated with the protein in solution for 3 h and 8 days respectively. EDX data for calcium fluoride (CaF_2_) present in the samples is also given*.

**Figure 4:**
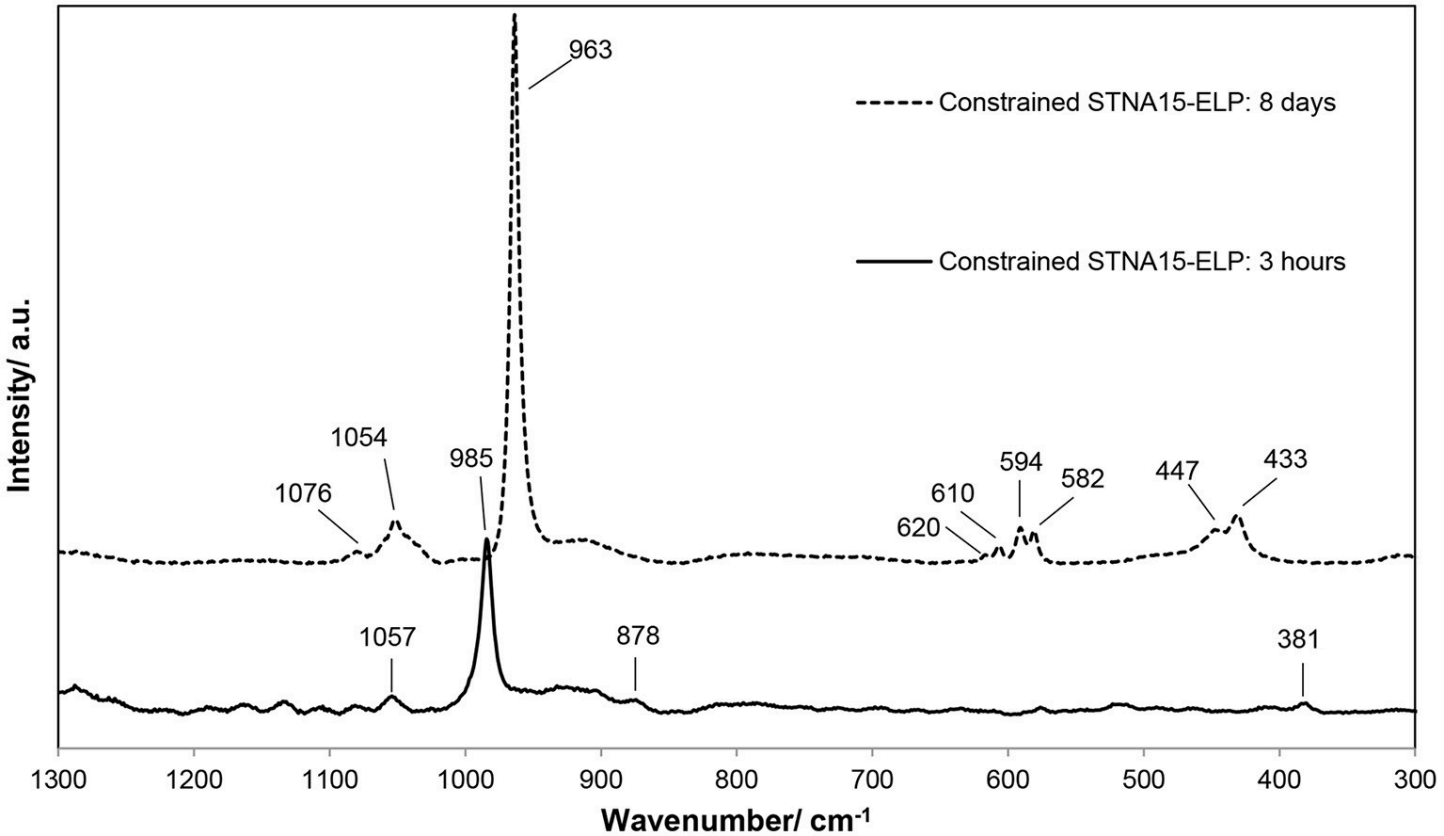
Raman spectroscopic data, in the range of 300–1,300 cm^−1^, of the mineral formed on the STNA15-ELP coated substrates at 3 h and 8 days of incubation.

The Raman spectra of the mineral formed after 3 h (Figure 4) exhibited a strong, sharp peak at 985 cm^−1^, typical of the *v*_2_ bending mode of ${\text{PO}}_{4}^{3-}$ in brushite. The peaks at 381 cm^−1^ (*v*_8_), 878 cm^−1^ (*v*_3_) and 1057 cm^−1^ (*v*_6_) are also indicative of brushite (Casciani and Condrate, 1979; Kim et al., 2002), supporting the SAED and EDX data. The Raman spectrum, of the mineral present in the constrained STNA15-ELP sample after 8 days of incubation, is that of apatite. Raman spectra of apatite are easily characterized by the strong, sharp peak at 961 cm^−1^, the *v*_1_ stretching mode of the ${\text{PO}}_{4}^{3-}$. Other indicative peaks are visible at 447 and 433 cm^−1^ from the *v*_2_ bending mode of the ${\text{PO}}_{4}^{3-}$; 620, 610, 594, and 582 cm^−1^ form the *v*_4_ bending mode of the ${\text{PO}}_{4}^{3-}$; 1076 and 1054 cm^−1^ from the *v*_3_ stretching mode of ${\text{PO}}_{4}^{3-}$.

**Figure 5:**
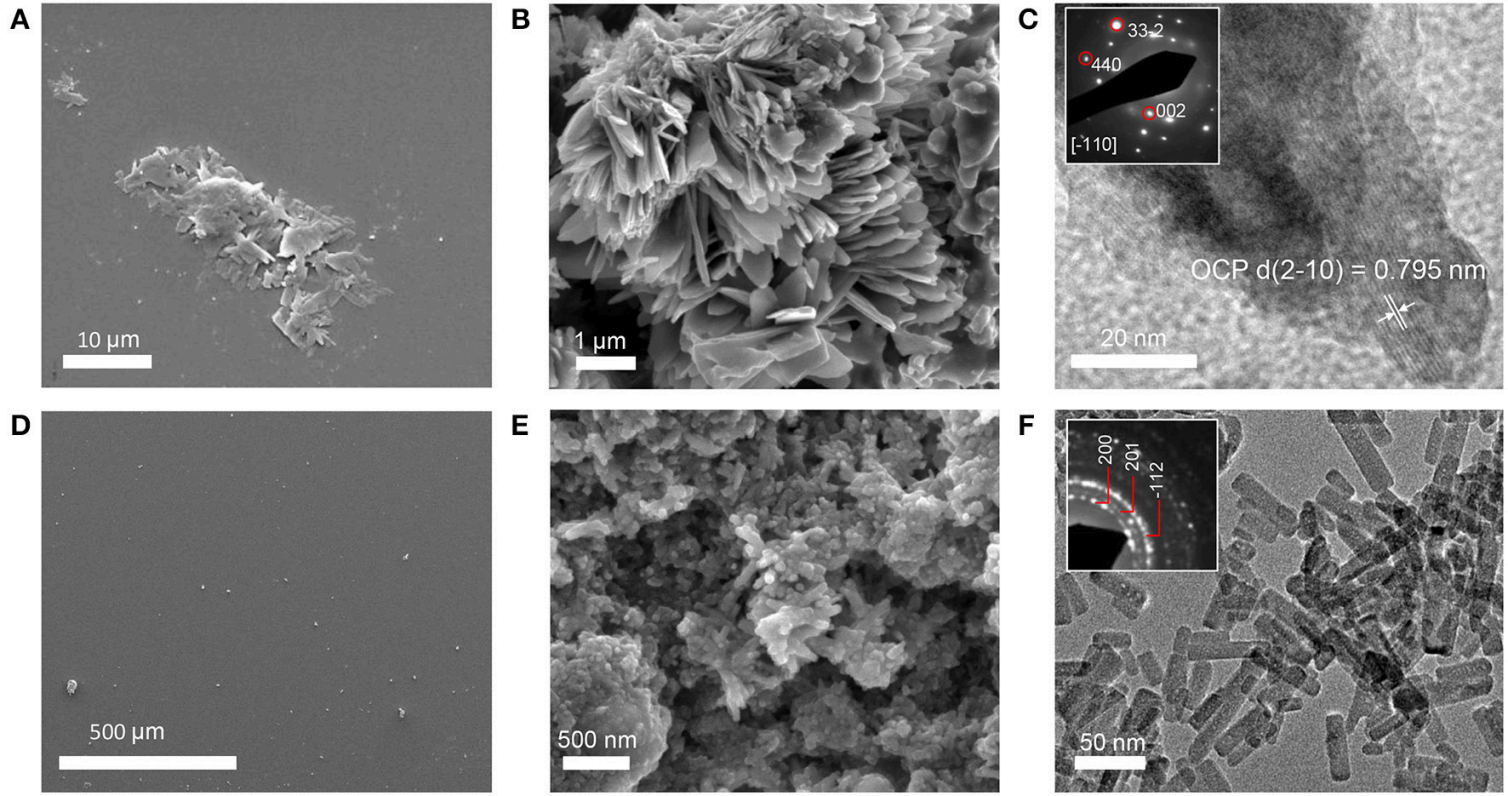
Precipitates formed in control conditions. **(A)** SE image of 3 h non-coated borosilicate glass. **(B)** SE image of precipitate formed in solution without STNA15-ELP. **(C)** High resolution TEM image of precipitate formed in solution with no protein and inset of C showing a typical SAED pattern of the precipitate. **(D)** SE image of the borosilicate surface after 8 days of incubation in mineralizing solution with no platelets present. **(E)** SE image of the precipitate in the control solution with no STNA15-ELP at 8 days. **(F)** TEM image of the precipitate formed in the control solution at 8 days with the inset showing a typical SAED pattern.

SE SEM images of the control sample, uncoated borosilicate glass, show that some mineral is present on the surface after 3 h of incubation (Figure 5A). This mineral resembled the shape of the brushite platelets seen in the STNA15-ELP coated samples. However, it appeared to be almost completely dissolved. In contrast to the coated borosilicate glass slide, after 8 days of incubation no mineral platelets were seen on the uncoated substrate (Figure 5D). The early dissolution of the mineral and the lack of mineral at the later time suggest that the ELP plays a critical role in stabilizing the early mineral phase.

SEM, EDX and Raman indicate that brushite was the first phase formed in the presence of the constrained STNA15-ELP coating and FAp was present after 8 days of incubation. For instance, brushite typically forms under acidic conditions (Dorozhkin, 2010), such as in this study (pH 6), explaining its presence at early time periods. Even though brushite seems to be the precursor to apatite in this study, brushite and FAp have different crystal structures and therefore are not likely to transform from one to another. Brushite has been reported to have a monoclinic structure (Sainz-Díaz et al., 2004) compared to the hexagonal FAp (Elliott, 1994). These different crystal structures lead to the conclusion that the change in mineral chemistry and morphology observed between the 3 h and 8 day period, coupled with the etch marks on the brushite surface, occurred due to a dissolution and re-precipitation process. The re-precipitated FAp crystallites were templated by the original brushite crystal, forming a layered ordered structure.

#### STNA15-ELP in solution

**Figure 6:**
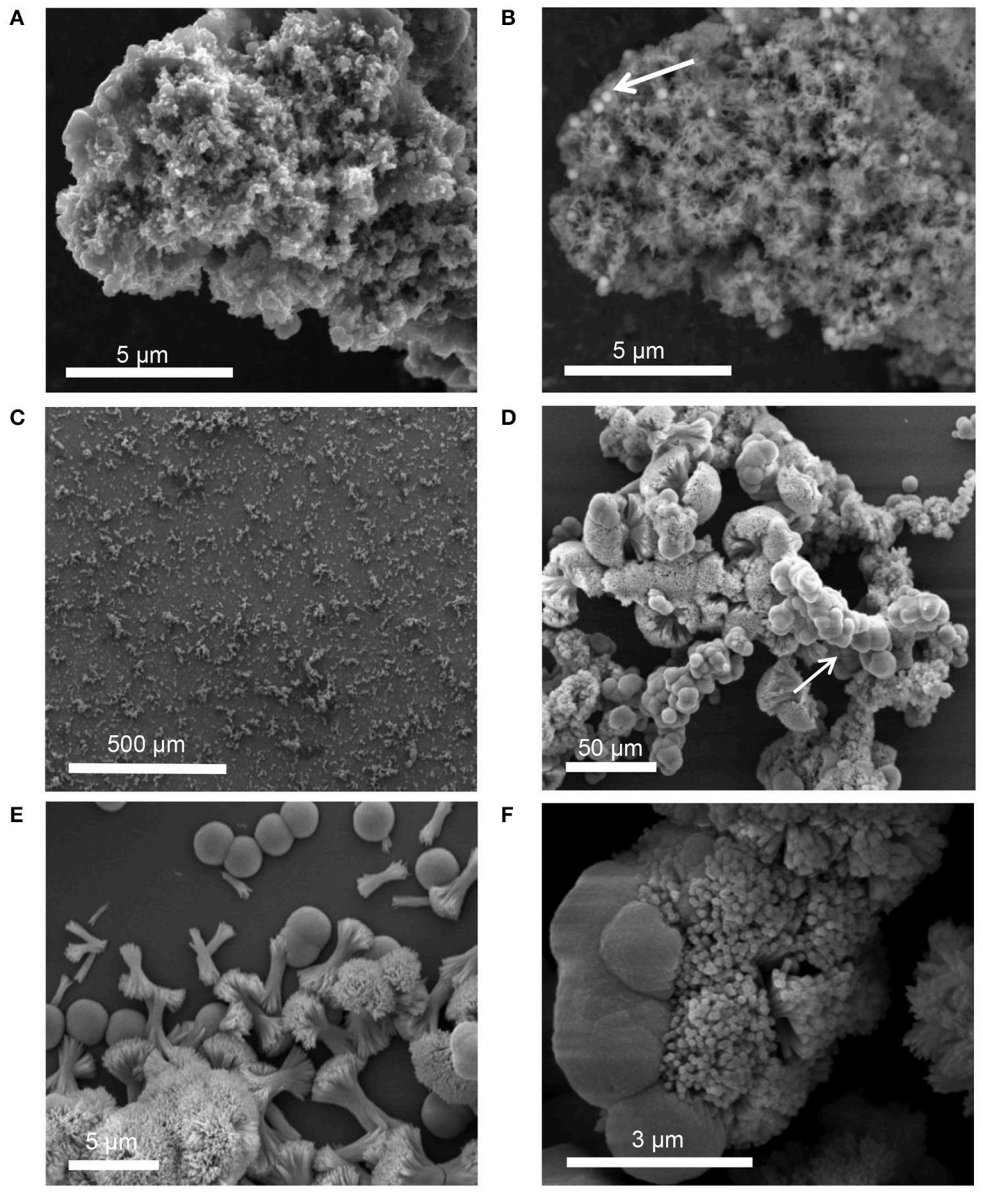
SEM images of precipitate formed in solution containing STNA15-ELP at 3 h [**(A)** SE and **(B)** BSE] and 8 days (SE: **C–F**) of incubation.

SE-SEM images of the lyophilized precipitate, formed with STNA15-ELP, are shown in Figure 6. After incubation for both 3 h (Figures 6A,B) and 8 days (Figures 6C–F), needle-like precipitates were visible. However, after 3 h of incubation the needles were only visible when imaged with BSE since they appeared to be buried within the protein (Figure 6B). EDX of the 3 h STNA15-ELP precipitate had a Ca/P ratio of 1.48 (3 hS, Table 3). After incubation for 8 days (Figures 6D–F) an abundance of spherical and dumbbell structures were observed and the EDX of the mineral suggested that a mixture of calcium fluoride (CaF_2_) (CaF_2_ in Table 3, Figure 6D arrow) and fluorapatite (8 dS in Table 3) existed. CaF_2_-like material has been previously reported to occur in solutions with high fluoride content, such as the one used in this study (Christoffersen et al., 1988; Mohammed et al., 2014). The EDX of the needles present in the 8 day sample gave a Ca/P ratio of 1.55. Since the mineral had a typical FAp needle-like morphology (for example Chen et al., 2006), the non-stoichiometric Ca/P ratio indicates a calcium deficiency in the apatite, analogous to previous literature (Dorozhkin, 2010). Also, the random orientation of the crystals, in both the 3 h and 8 day samples, indicates that the nucleation process in solution is spontaneous and the apatite has no preferred growth direction.

**Figure 7:**
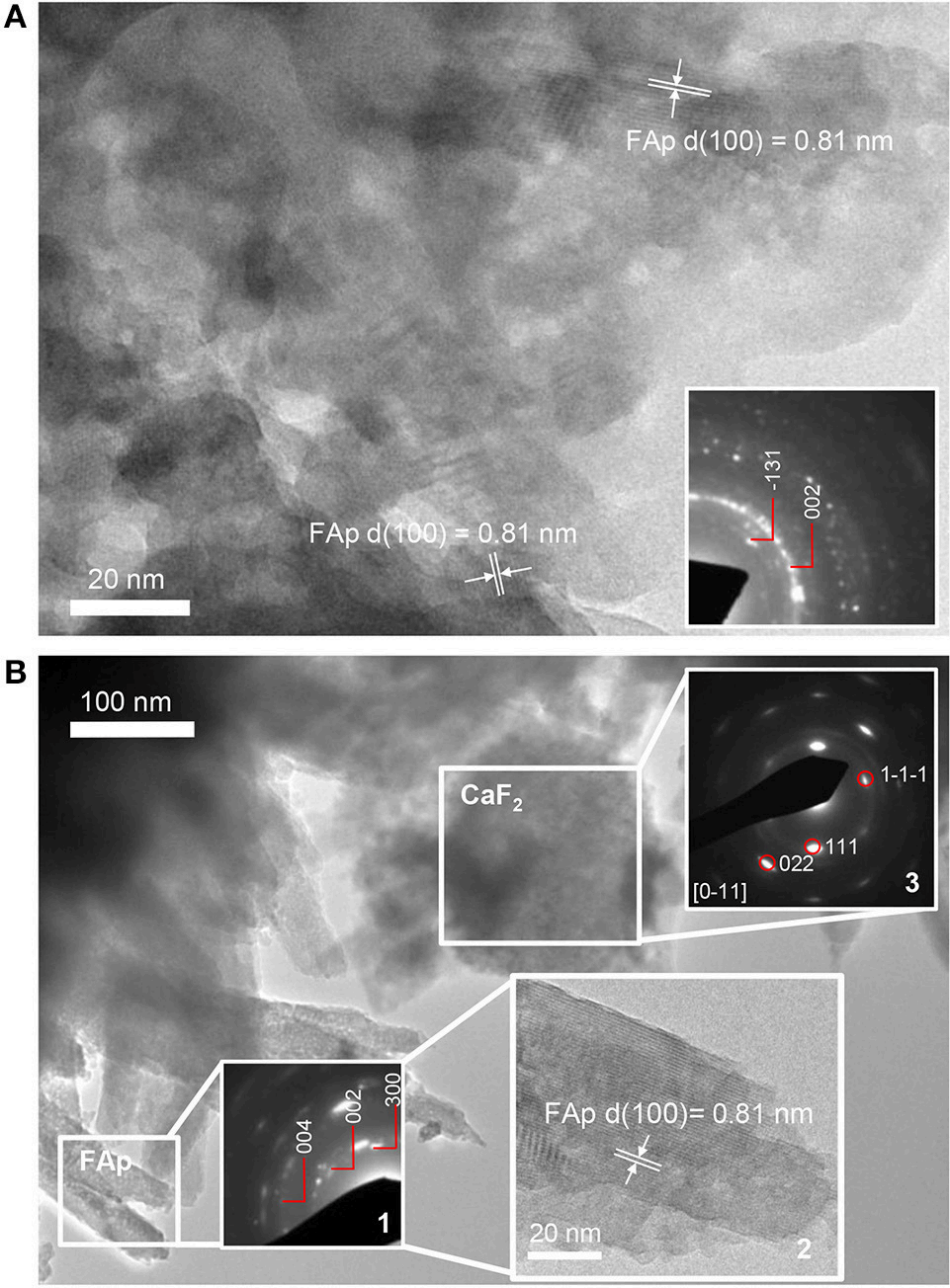
TEM images of precipitate present in solution containing STNA15-ELP at **(A)** 3 h and **(B)** 8 days of incubation (inset 2 showing the high resolution TEM image with FAp planes visible). The 3 h precipitate is a nano-crystalline apatite. At 8 days the apatite has a needle-like form. The needles that were directly in contact had some co-alignment as seen in the SAED pattern (inset 1). The spherical particles, present at 8 days, were confirmed to be CaF_2_, from the SAED pattern (inset 3).

TEM of the 3 h precipitate, formed in the presence of STNA15-ELP, showed nano-crystalline mineral. The high resolution TEM (Figure 7A) and SAED (inset of Figure 7A) of the early precipitate both indicate FAp was already present at 3 h. The 8 day precipitate was also confirmed to be FAp, both with SAED (inset 1 of Figure 7B) and high resolution TEM (inset 2 of Figure 7B). The SAED of the spherical particles confirmed the presence of calcium fluoride (inset 3 of Figure 7B).

**Figure 8:**
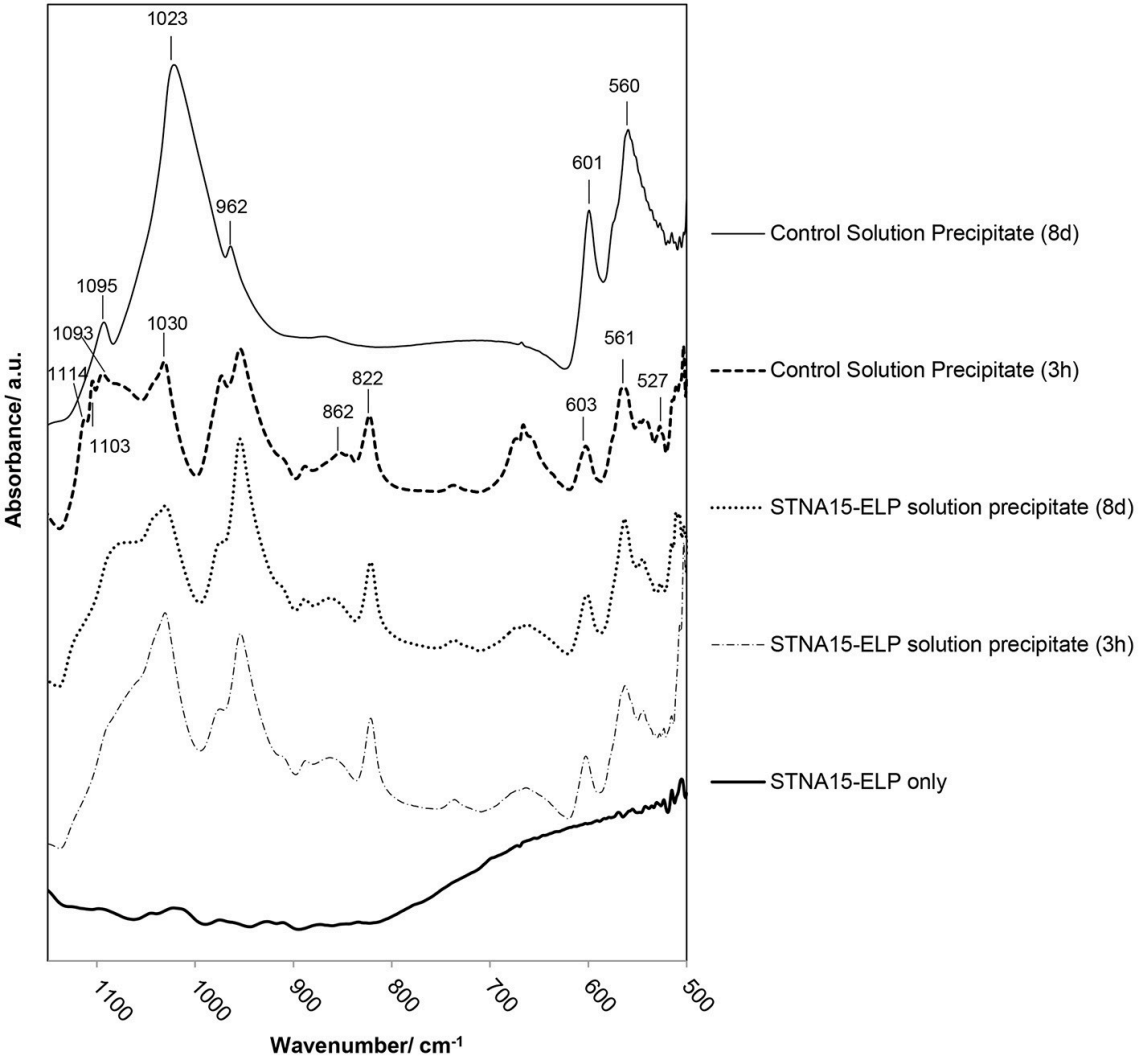
FTIR analysis of precipitate formed in solution with and without STNA15-ELP. Without STNA15-ELP OCP forms at 3 h, transforming to highly crystalline FAp at 8 days. The ELP promotes FAp formation, with the FTIR indicating the presence of nano-crystalline FAp at 3 h and 8 days. (820 cm^−1^: residual NO_3_).

FTIR analysis of precipitate formed in solution with free STNA15-ELP (Figure 8), after 3 h of incubation, shows broad and poorly defined peaks generated by the *v*_3_ asymmetric stretching mode of the apatitic ${\text{PO}}_{4}^{3-}$ group (1,000–1,100 cm^−1^). Further apatitic peaks, generated by the *v*_4_ bending mode of the ${\text{PO}}_{4}^{3-}$ group, are visible at 603 and 561 cm^−1^ (Kim et al., 2002). FAp and HAp are crystallographically identical and not distinguishable in many analytical techniques. However, FAp can be characterized in FTIR by the lack of the OH^−^ liberation peak at 631 cm^−1^, normally present in the HAp spectra. The traces in Figure 8 lack this liberation peak, indicating that the apatite precipitate is a fluorapatite. A peak indicative of the ${\text{HPO}}_{4}^{2-}$ group (527 cm^−1^) is present in the spectra of both the 3 h and 8 day precipitate formed in the presence of free STNA15-ELP, normally present in octacalcium phosphate (OCP).

FTIR of the control precipitate, formed after 3 h of incubation, has a typical OCP trace with peaks generated by the *v*_3_ ${\text{HPO}}_{4}^{2-}$ stretch visible at 1114, 1103 and 1093 cm^−1^, in addition to the 527 cm^−1^ peak that is visible in the protein containing samples (Berry and Baddiel, 1967; Fowler et al., 1993). SEM of the control precipitate at 3 h shows typical OCP platelets (Figure 5B) and its presence was further confirmed with high resolution TEM and SAED (Figure 5C and **inset of 5C**). The control precipitate found in the 8 day samples had a typical fluorapatite FTIR spectrum with well-defined apatitic peaks. The apatite crystallites of the control had typical hexagonal needle shapes, seen in SE SEM images (Figure 5E) and TEM images (Figure 5F). The needles had regular ends, unlike the needle-like precipitate formed in the presence of STNA15-ELP (Figure 7B).

The FAp crystals initially formed within an aggregate of STNA15-ELP, where favorable heterogeneous nucleation can occur. The broad peaks of the PO_4_ group, seen in the 3 h FTIR traces (Figure 8) of the precipitate formed in the presence of ELP, indicate that nano-crystalline apatite had formed, as observed in the TEM image (Figure 7A). The low Ca/P ratio of the 3 h precipitate was lower than calcium deficient apatite and was tending toward OCP. Furthermore, the presence of the ${\text{HPO}}_{4}^{2-}$ peak in the FTIR spectrum is contradicting the presence of apatite. Rey et al. have extensively analyzed nano-crystalline apatite with techniques such as FTIR, NMR and XRD (Eichert et al., 2004; Rey et al., 2007a,b; Drouet et al., 2009). Rey et al. have hypothesized that nano-crystalline apatite is imperfect and is surrounded by a hydration layer. Due to this hydration layer, nano-apatite has a striking likeness to OCP. Firstly, the nano-apatite Ca/P ratio is somewhere in between OCP and apatite, and increases with the maturation of the crystals (Drouet et al., 2009). Similarly, FTIR of nano-apatite can have non-apatitic characteristics, resembling OCP traces, explaining the presence of the ${\text{HPO}}_{4}^{2-}$ peak in the FTIR. The initial presence of nano-crystalline apatite can further explain the ragged shape of the crystals seen under TEM (Figure 7B). Rey et al. (2007b) have hypothesized that during maturation, the nano-crystals fuse together at the expense of the hydrated OCP-like layer.

### Protein conformation and immobilization affects route of mineral formation and mineral morphology

Differences were observed in the mineralization process and the FAp morphology obtained at the 8 day incubation period between the constrained and unconstrained STNA15-ELP mineralized samples. In the case of the constrained protein, the ordered STNA15-ELP on the glass surface appeared to restrict the formation of the FAp to polycrystalline platelet morphology. The results strongly suggest that there are two reasons for this. Firstly, the brushite platelets exist for longer periods of time in the presence of STNA15-ELP coating. Reports have shown that other proteins, such as bovine serum albumin, can retard the transformation of brushite to apatite (Xie et al., 2001, 2002). Although there is no clear explanation for this, it is speculated that the adsorbed protein prevents water molecules from making direct contact with the surface of the brushite and therefore prevents dissolution.

The second explanation is based on templated mineralization. The stabilized brushite crystals, surrounded by the protein, slowly dissolved once formed. The dissolution of the crystals was evidenced by the etch pits visible on the surface (Figure 3B). As brushite formed, along with the calcium fluoride precipitate, the calcium-pH isotherm shifts to an area where brushite was no longer stable, causing dissolution. The brushite dissolution created a local supersaturation of calcium that could reprecipitate as FAp, in the presence of fluoride. The slow FAp formation process allowed an ordered structure to form where the FAp crystallites grow along the {010} face of brushite.

A different process occurred when the protein was unconstrained and free in solution. As seen in Figure 3B, the nucleation was random and the growth of the crystals had no preferred orientation. The protein aggregates created an environment which favored apatite formation over other phases, even at 3 h of incubation. This may be due to local buffering effects caused by the lysine amino acids in the STNA15-ELP sequence. Since the lysine groups are likely to interact with the borosilicate surface, this effect is not seen with the STNA15-ELP coating. Other mineralizing organic molecules, such as amelogenin, have shown capability of buffering pH and stabilizing precursor phases. Amelogenin has the ability to stabilize amorphous calcium phosphate in a solution which normally precipitates apatite (Kwak et al., 2011).

The specific behavior observed in this study can be further supported by the thermoresponsive properties of ELPs related to their ITT. ELPs are known to fold and aggregate above their transition temperature. In its folded state, the STNA15-ELP can display, when in solution, its nucleating sites on the surface causing FAp nucleation all over the aggregate. The nucleation on ELP aggregates is consistent with other work where needle like apatite grew from spherical ELP particles (Misbah et al., 2016). However, once adsorbed on glass and with an increase in order, the STNA15 sequence of the ELP is no longer concentrated on a surface of a particle but on a flat substrate. In both cases the mineral formation resembled the process of biomineralization where either a precursor phase was present before a stable mineral is formed (for example Johnsson and Nancollas, 1992) or smaller sub-units fused to form larger, imperfect crystals (for example Robinson, 2007). However, only the constrained protein produced mineral that has a preferential growth direction.

These findings provide information that can lead to synthesis of ordered FAp structures that could be used in enamel therapeutics. However, more importantly, this synthetic biomineralizing system has shown that protein constraint and protein conformation both play an extremely important role in the process of biomineralization. Protein constraint can be related to natural biomineralizing processes, where the mineralizing proteins exist within a gel-like matrix. In fact proteins, such as amelogenin, have been shown to assemble into fibers and nanospheres, rather than existing in a free form (Carneiro et al., 2016).

## Conclusion

This study has demonstrated the ability of the STNA15-ELP to control the route of formation of FAp and its subsequent morphology. The morphological difference of FAp observed in the two conditions can be attributed to the STNA15-ELP conformation and interaction with a substrate. The addition of STNA15-ELP to a fluoridated mineralizing solution yielded FAp when the protein was both constrained on a surface and free in solution. However, plate-like stoichiometric FAp was found on protein coated borosilicate compared to needle-like calcium deficient FAp with the protein un-constrained in solution. The plate-like FAp was templated by pre-existing brushite platelets forming structures which were organized in a particular direction. The multistep FAp formation presented here resembles the natural biomineralization process, where transient metastable phases precede the final stable calcium phosphate. Ordered FAp formation is preferred for use in enamel therapeutics and therefore the constrained protein will be pursued in further studies in order to optimize and control the mineralization process.

## Author contributions

KS, carried out the data collection, data analysis and interpretation and drafted the article; MA, PA, AM, HSA, and AB, are project supervisors and all took part in the critical article revision; MA, PA, and AM, were the initial conception designers; NT, carried out the TEM work and critical revision of the article; Final approval of the article by MA.

### Conflict of interest statement

The authors declare that the research was conducted in the absence of any commercial or financial relationships that could be construed as a potential conflict of interest. The reviewer YZ and handling Editor declared their shared affiliation, and the handling Editor states that the process nevertheless met the standards of a fair and objective review.

## Abbreviations

STNA15: The analog of the 15 amino acid *N*-terminal of natural human statherin protein
ELP: Elastin-like protein
STNA15-ELP: Elastin-like protein containing the analog of the 15 amino acid *N*-terminal of statherin.
